# Synthesis of amantadine clubbed *N*-aryl amino thiazoles as potent urease, α-amylase & α-glucosidase inhibitors, kinetic and molecular docking studies†

**DOI:** 10.1039/d3ra05330j

**Published:** 2023-08-22

**Authors:** Fatima Tuz Zahra, Aamer Saeed, Atteeque Ahmed, Hammad Ismail, Muhammad Umar Ijaz, Fernando Albericio

**Affiliations:** a Department of Chemistry, Quaid-i-Azam University 45320 Islamabad Pakistan asaeed@qau.edu.pk +92-51-9064-2241 +92-51-9064-2128; b Department of Biochemistry and Biotechnology, University of Gujrat Gujrat 50700 Pakistan; c Department of Zoology, Wildlife and Fisheries, University of Agriculture Faisalabad 38040 Pakistan; d Peptides Science Laboratory, School of Chemistry and Physics, University of KwaZulu-Natal Westville Durban 4000 South Africa; e CIBER-BBN, Networking Centre on Bioengineering, Biomaterials and Nanomedicine, Department of Organic Chemistry, University of Barcelona 08028 Barcelona Spain

## Abstract

A series of ten novel compounds were synthesized by incorporating a 1,3 thiazole core into amantadine and their structures were validated using different analytical and spectral methods such as FTIR, EI-MS, ^1^H NMR, and ^13^C NMR. The antibacterial and enzyme inhibitory properties of these newly synthesized compounds were evaluated. Remarkably, the compounds exhibited significant antibacterial activity against *Escherichia coli* and *Bacillus subtilis*. Additionally, the *in vitro* inhibitory activities of the synthesized compounds, against α-amylase, α-glucosidase, and urease were investigated. Among the tested compounds, compound 6d demonstrated potent and selective inhibition of α-amylase IC_50_ = 97.37 ± 1.52 μM, while acarbose was used as positive control and exhibited IC_50_ = 5.17 ± 0.25 μM. Compound 6d and 6e exhibited prominent inhibition against α-glucosidase IC_50_ = 38.73 ± 0.80 μM and 41.63 ± 0.26 μM respectively. Furthermore, compound 6d inhibited urease with exceptional efficacy IC_50_ = 32.76 μM, while positive control thiourea showed more prominent activity having IC_50_ = 1.334 μM. Molecular docking studies disclosed the binding mechanism and affinity of these new inhibitors within the binding sites of various amino acids. To investigate the association between molecular structural characteristics and inhibitory actions of synthesized derivatives, preliminary structure–activity relationship (SAR) studies were performed. These findings indicated that compounds 6a, 6c, 6d and 6e are potential candidates for hit-to-lead follow-up in the drug-discovery process for treating diabetes and hyperglycemia.

## Abstract 2

A series of ten novel compounds were synthesized by incorporating a 1,3 thiazole core into amantadine and their structures were validated using different analytical and spectral methods such as FTIR, EI-MS, ^1^H NMR, and ^13^C NMR.

## Graphical abstract

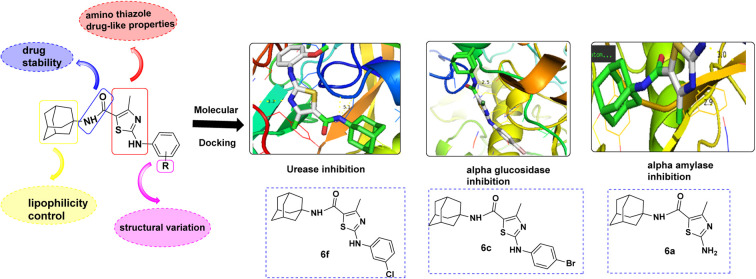

## 1. Introduction

Heterocyclic compounds possess tremendous potential as key structures in drug discovery and development due to their ability to interact with a wide variety of molecular targets and participate in a diversified array of binding interactions.^1^ Due to their therapeutic potency, lipophilicity, and other properties, thiazoles are extremely useful substructures in the pharmaceutical industry.^2^ Thiazoles motifs are biologically significant five-membered heterocycles, possessing various activities such as anti-cancer,^3^ anti-tubercular,^4^ antimicrobial,^5^ analgesic, and anti-inflammatory^6^ bioactivities, and are just a few of the thiazole-containing compounds. Thiazole heterocycles have also demonstrated efficacy in diverse applications such as bacteriostatic,^7–9^ antibiotics,^10^ antifungal,^11^ CNS regulants of high selling diuretics,^12^ local anesthetics,^13^ HIV infections,^14,15^ anti-allergic,^16^ antihypertensive,^17^ against schizophrenia,^18^ anti-diabetic,^19^ and anthelminthic.^20^ Owing to their electron rich nature, they are ideal candidates for non-covalent interactions with enzymes or receptors. They have also been employed in materials chemistry due to their optical or electronic properties.^21^ Moreover, the thiazole ring is found in numerous highly active substances. For instance, Meloxicam, a new nonsteroidal anti-inflammatory medication (NSAID), includes a thiazolyl group in its structure. Other thiazole derivatives, such as Niridazole and Ritonavir, have been found to be effective drugs for ulcer therapy and antiretroviral treatments, respectively. This emphasizes the significant role of thiazole-containing compounds in the synthesis of therapeutically beneficial molecules.

Amantadine (1-aminoadamantane) works as a nicotinic antagonist and a noncompetitive *N*-methyl-d-aspartate (NMDA) antagonist, making it useful in the treatment of Alzheimer's disease.^22^ Different amino acetyl adamantyl amines can inhibit the Vaccinia virus,^23^ and 1-adamantane carboxylic acid amides can act as potential inhibitors for the smallpox virus and, more recently, several coronaviruses.^24^ The compounds bearing adamantly substituent have demonstrated their effectiveness in the treatment of disease conditions as anti-viral and anti-type 2 diabetic agents. The steric bulk of the adamantyl group has the ability to either limit or moderate intramolecular reactivity, as well as to prevent hydrolytic enzymes from entering the drug, thereby increasing plasma half-life and drug stability.^25^

The amide linkage is one of nature's most important functional groups because it constitutes the backbone of all natural peptides and proteins. This carboxamide linkage is vital in determining a drug stability, pharmacokinetics, and pharmacodynamics. Amide linkage is highly stable under physiological conditions which is essential for a drug to withstand the harsh environment of the body. It ensures that the drug remains intact during storage and metabolism allowing it to reach it target site without degradation. Hence, it enhances drug potency facilitating drug–target interactions. The presence of an adamantyl group, amide linkage, a heterocycle thiazole, and an alkyl and aryl substituent for structural variation are essential structural features of our designed molecules. It was speculated, based on the literature review, that a hybrid pharmacophoric method could be incorporated to synthesize novel molecules with a diverse pharmacological property. As a result, molecular hybridization was adopted to unite the three scaffolds together into single structural unit with a synergistic impact on all.

In this study, a series of new condensed heterocyclic compounds with various substituted phenyl moiety and bridgehead amantadine fused thiazoles were designed and evaluated for their inhibition potential in suppressing hyperglycemia and diabetes. Previous studies primarily focused on these types of compounds for single target bioactivity, but our research aimed to explore the multi-target biological potential of title compounds. Thiazole and amantadine are well recognized medicinally active heterocyclic units, and this prompted us to explore molecules constituting these two motifs and seek their multitarget biological potential. To a step further, molecular docking studies were performed to define the models for comprehension of binding interactions and to delineate the binding affinity of the molecules in the active sites of target proteins.

α-Amylase and α-glucosidase enzymes are critical glycoside hydrolases that play a crucial role in carbohydrate digestion. These enzymes are primarily found in the cells lining the small intestine. In the small intestine, α-amylase breaks down polysaccharides into disaccharides, while α-glucosidase is responsible for breaking down α-glucopyranosides bonds in polysaccharides and oligosaccharides into monosaccharides such as fructose and glucose. α-Glucosidase also contributes to the final phase of carbohydrate digestion.^26^

Diabetes is a prevalent metabolic disease affecting over 400 million people worldwide. This disease results in disturbances in carbohydrate, protein, and fat metabolism due to hormone secretion and insulin deficiency.^27^ Early diagnosis and appropriate treatment methods are crucial to reducing the burden of diabetes on both individuals and society. As a result, research on the suppression of diabetic complications is gaining importance.^28^ One of the most effective ways to control diabetes and prevent related complications is to regulate blood glucose levels.^29^ The use of α-amylase and α-glucosidase inhibitors as part of diabetic treatment is a viable option. These inhibitors can help restore blood glucose to normal levels by inhibiting the activity of these digestive enzymes.^30^ Therefore, various newly synthesized inhibitor forms associated with urease, α-amylase, and α-glucosidase enzymes are critical in the treatment of diseases such as glaucoma, obesity, epilepsy, cancer, and diabetes.

**Fig. 1:**
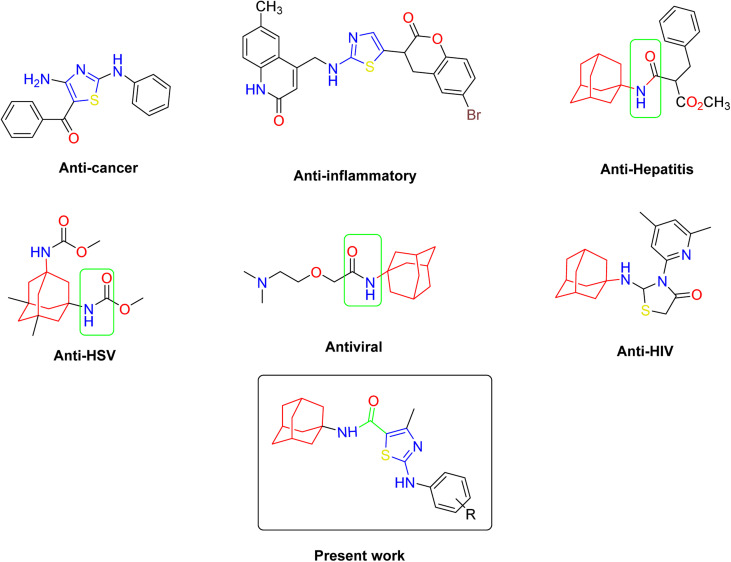
Some pharmacologically active compounds possessing amantadine, thiazole and carboxamide linkage.

Similarly, the urease enzyme is a major focus of interest for biochemists, physiologists, and medicinal chemists due to its role in causing stomach cancer and peptide ulceration when produced by certain bacteria.^31^ Additionally, a buildup of urea in the body can lead to uremia, metabolic acidosis, high blood pressure, swelling, and skin problems. This hydrolysis process is catalyzed by a nickel-containing enzyme called urease, which is found in various bacteria and performs the hydrolysis of urea at an incredibly high rate compared to an uncatalyzed reaction.^32–34^ However, mammalian cells do not produce urease, and its activity can lead to medical issues such as urinary stones, catheter blockage, pyelonephritis, and hepatic coma.^35^ To combat these issues, various strategies have been developed to inhibit the activity of bacterial ureases. The goal of this study is to evaluate the effects of newly synthesized derivatives on the activity of urease, α-glucosidase, and α-amylase enzymes (Fig. 1).

## 2. Results and discussion

### 2.1 Chemistry

**Scheme 1:**
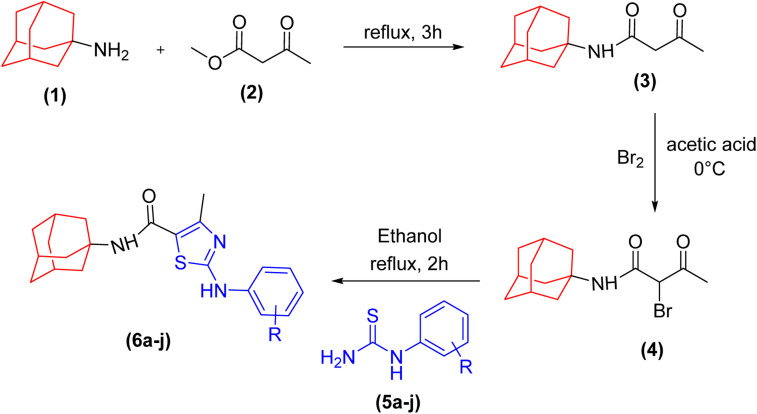
General synthetic scheme of amantadine-based *N*-aryl amino thiazoles.

A series of novel amantadine clubbed *N*-aryl amino thiazoles have been prepared according to route shown in (Scheme 1). The first step involves the synthesis of adamantyl oxobutanamide by nucleophilic substitution reaction. Then, the resultant amide was treated with bromine to furnish adamantyl bromo oxobutanamide in an excellent yield. Finally, the cyclization of resultant bromo compound is done by treating it with differently substituted hydrolyzed thioureas in dry ethanol.^36,37^

**Fig. 2:**
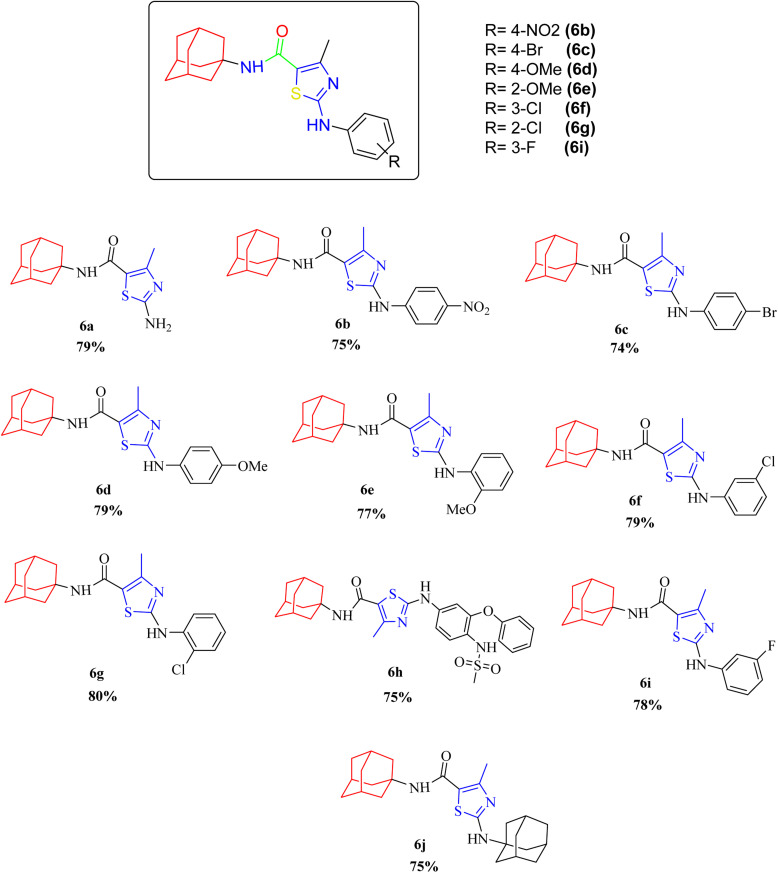
Chemical structures and % yields of synthesized compounds (6a–j).

Compound 6c has bromine substituent on *para* position of phenyl ring and shows a moderate yield of 74% when compared to other derivatives. Compound 6g was produced with an excellent yield of 80%, the highest yield of any compound. Overall, this is a multistep synthetic approach, and all of the steps involved are clean and high yielding. The products appeared as a single spot on the TLC plates, and there was no prerequisite to use column chromatography to separate the products (Fig. 2).

The compounds were fully characterized by physical techniques. The synthesis of new amantadine fused thiazoles was indicated in the FTIR spectra by the presence of two strong peaks between 3424 cm^−1^ and 3100 cm^−1^ was assigned to NH of amide and amine linkage. The peaks around 1560 cm^−1^ (indicating presence of aromatic ring) and the absorption bands for C–N appeared at 1470 cm^−1^. The Csp^3^–H stretching of alkyl part appeared as 2 peaks between 2700 to 2800 cm^−1^. Presence of carbonyl functional group was confirmed by appearance of absorption peak around 1700 cm^−1^ indication presence of amide linkage in the structure. The synthesis of compounds was further confirmed by ^1^H NMR spectra. The protons in the vicinity of electron withdrawing groups (NO_2_, F) appeared more deshielded as compared to the electron donating groups (OMe). In the aromatic part of the spectrum multiplet were observed for monosubstituted rings and doublets of doublets were observed for *para*-substituted rings. The methyl groups appeared at 2.96 ppm which is consistent with their environment being directly attached with thiazole ring. The ^13^C NMR spectra demonstrated that ipso carbons were found relatively deshielded and the same electronic effect was observed for carbons attached with electron donating or electron withdrawing groups. The downfield absorption of OCH_3_ carbon relative to methyl carbon can be attributed to electronegativity effect exerted by oxygen atom in the case of the latter.

The characteristic amine proton appeared at a value between 10.5 and 11.9 ppm. The phenyl ring was attributed to the presence of signals between 7–8 ppm. The proton signals for amide and azomethine appeared at approximately 8 ppm and 6–7 ppm, respectively. The sp^3^ carbons of amantadine exhibit a modest variation in their chemical environment, with a value between 2 and 3 ppm. The ^13^C-NMR spectrum of compounds indicated the presence of isochronous carbons. The carbon of the amide moiety appeared between 165 and 170 ppm. Aryl ring signals appeared between 120 and 140 ppm, whereas the signals between 12 and 28 ppm attributed to sp^3^ carbons of alkyl region.

### 2.2 Biological studies

The synthesized compounds were subjected to three different activities along with anti-microbial activity. All the compounds showed great inhibition against three enzymes and α-amylase, α-glucosidase and urease. In case of α-amylase, selected compounds exhibited higher inhibition potential than acarbose. Particularly, compound 6d was the most active compound in the series due to presence of methoxy group at the *para* position which resulted in the donation of electron density through no-bond resonance. The compounds showed significant inhibition against α-glucosidase enzyme, and derivative 6c was found to be the most potent compound in the series due to possession of halogen atoms at *para* position of phenyl ring. 6c contains bromine atom at *para* position. Moreover, some derivatives were found potent against urease enzyme and compound 6d showed better results in the series. 6d contained –OMe group at the *para* position of phenyl ring which served as electron donator site.

#### 2.2.1 Bioactivity results

##### 2.2.1.1 Antibacterial assay

Anti-bactericidal activity of the compounds was determined by using disc diffusion method. Results were measured in terms of zones of inhibition zones and are presented in Table 1. Results showed that 6a has significant antibacterial activity against all tested strains. On the other hand, 6c and 6g showed activity against one bacterial strain out of four. However, 6b, 6d, 6e, 6f showed no antibacterial activity against any tested bacterial strains. The assay was performed in triplicate and Kanamycin was used as positive control. In contrast to molecules containing electron donating groups, Chikhalia *et al.*^38^ found that the presence of multiple electron withdrawing groups generally boosted the antibacterial property. According to Yildiz *et al.*^39^ the antibacterial activity of a substitution connected to a biologically active molecule may be increased.

**Table 1:** Results of biological evaluation of TH series^a^

Name	Antibacterial zone of inhibition				DPPH	α-Amylase	α-Glucosidase	Urease
	*Escherichia coli*	*Pseudomonas aeruginosa*	*Bacillus subtilis*	*Staphylococcus aureus*	IC_50_ μM			
6a	11 mm	10 mm	13 mm	8 mm	157.4 ± 10.30	118.3 ± 0.71	72.12 ± 0.11	86.65 ± 0.22
	MIC = 50 μM	MIC = 50 μM	MIC = 50 μM	MIC = 75 μM				
6b	-	-	-	-	107.8 ± 1.93	-	-	43.45 ± 0.59
6c	-	-	7 mm	-	126.4 ± 2.66	-	98.65 ± 0.70	49.28 ± 1.45
			MIC = 75 μM					
6d	-	-	-	-	19.19 ± 0.37	97.37 ± 1.53	38.73 ± 0.80	32.76 ± 0.28
6e	-	-	-	-	110.3 ± 0.76	-	41.63 ± 0.26	48.48 ± 0.81
6f	-	-	-	-	136.7 ± 1.33	-	-	-
6g	-	-	7 mm	-	121.6 ± 6.54	-	-	127.2 ± 2.62
			MIC = 75 μM					
6h	-	-	-	-	64.1 ± 0.71	-	-	64.97 ± 0.67
6i	-	-	-	-	131 ± 1.11	-	-	112.20 ± 1.70
6j	-	-	-	-	137.9 ± 0.90	-	-	-
Kanamycin	20 mm	22 mm	21 mm	24 mm		-	-	
	MIC = 25 μM	MIC = 20 μM	MIC = 25 μM	MIC = 20 μM				
Ascorbic acid	-	-	-	-	0.89 ± 0.37	-	-	-
Acarbose	-	-	-	-	-	5.17 ± 0.25	1.21 ± 0.16	-
Thiourea	-	-	-	-	-	-	-	1.34 ± 0.29

a “-” in table refers to compounds that are inactive for respective enzyme inhibition.

##### 2.2.1.2 DPPH assay

Antioxidant potential of compounds were investigated using DPPH assay and results in the form of IC_50_ values are presented in Table 1. Ascorbic acid was used as positive control which exhibited strong activity with IC_50_ value 0.892 ± 0.36 μM. The results of IC_50_ represented that the highest activity was shown by the compounds 6d with IC_50_ value 19.19 ± 0.37 μM followed by the compound 6h with IC_50_ value 64.1 ± 0.7 μM. Compounds 6a, 6b, 6j and 6i showed comparatively lower activity with IC_50_ range 130 μM to 157 μM while rest of the compounds exhibited moderate DPPH scavenging activity with IC_50_ range from 80 to 120 μM. Overall, compounds exhibited moderate to good antioxidant potential. Alkan *et al.*^40^ investigated the effects of many novel 3,4-disubstituted, 4,5-dihydro-1*H*-1,2,4-triazol-5-one derivatives and discovered that, depending on concentration, all of the compounds exhibited DPPH free radical scavenging properties. According to Meng *et al.*'s^41^ paper from 2010, substitution on the benzene ring increases the ability to donate electrons, which is advantageous for boosting antioxidant activity.

##### 2.2.1.3 α-Glucosidase inhibition assay

Newly synthesized compounds were tested for their ability to inhibit α-glucosidase enzyme and results in the form of IC_50_ values are presented in Table 1. Acarbose was used as positive control which showed prominent enzyme inhibition activity with IC_50_ value 1.21 ± 0.16 μM. The results of IC_50_ represented that the highest activity was shown by the compound 6c with IC_50_ values 38.73 ± 0.80 μM followed by the compounds 6e, 6a and 6c with IC_50_ value 41.63 ± 0.26, 72.12 ± 0.11 and 98.65 ± 0.70 μM respectively. Overall, compounds exhibited good α-glucosidase inhibition potential. After enzyme inhibition studies compounds were subjected to docking with protein receptors of α-glucosidase and results of interactions are presented in Fig. 4 and Table 2. Among these ligands 6a, 6b and 6e showed the strongest interaction with the lowest energy as −3.6, −0.7, and −1.5 kcal mol^−1^ respectively. These compounds also showed dual interaction with α-glucosidase receptor at amino acid ARG-609, ARG-587, and THR-384 having interaction distance between 2.5 to 3.5 Å (Table 2). These results represent strong positive interactions of compounds with enzyme which might be possible for the *in vitro* biological activity. In a variety of metabolic pathways, such as the processing of glycoproteins and glycolipids and the intestinal digestion of carbohydrates, glycoside trimming enzymes play a critical role. Since they catalyze the release of glucose from the non-reducing end of an oligo- or polysaccharide chain involved in glycoprotein production, glucosidases are thought to be a potent therapeutic target among the wide variety of enzymes.^42^

**Table 2:** Results of docking studies^a^

Sample	Binding energy (kcal mol^−1^)			Binding residues/bond lengths (Å)		
	α-Glucosidase	α-Amylase	Urease	α-Glucosidase	α-Amylase	Urease
6a	−3.6	−6.1	−5.2	Arg-609 = 3.5	Ser-4 = 2.9, Thr-6 = 3.0	Arg-145 = 3.1
				1 interaction	2 interactions	1 interaction
6b	-	-	−6.1	-	-	Arg-141 = 3.1 and 3.0
						2 interactions
6c	−0.7		−5.6	Thr-384 = 2.5	-	Asn-16 = 3.5
				1 interaction		1 interaction
6d		−1.1	−5.6	-	Arg-420 = 2.8	Tyr-143 = 3.0
					1 interaction	1 interaction
6e	−1.5	-	−6.1	Arg-587 = 3.1	-	Asn-16 = 5.1, Arg-142 = 3.3
				1 interaction		2 interactions

a “-” in table refers to compounds that are inactive for respective enzyme inhibition.

##### 2.2.1.4 α-Amylase inhibition assay

**Fig. 3:**
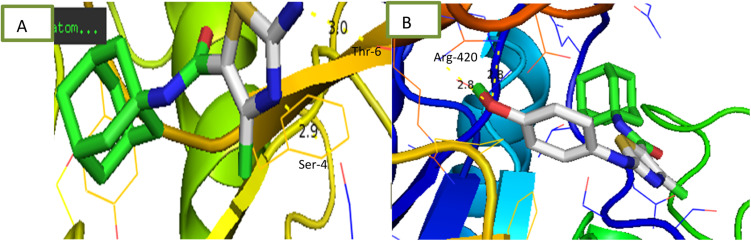
Interaction of α-amylase with (A) 6a (B) 6d.

Compounds were tested for their ability to inhibit α-amylase enzyme and results in the form of IC_50_ values are presented in Table 1. Acarbose used as positive control which exhibited prominent enzyme inhibition activity IC_50_ value 5.17 ± 0.25 μM. The results of IC_50_ represented that the compounds 6a and 6d showed activity with IC_50_ value 118.3 ± 0.71 and 97.37 ± 1.53 μM. Only these two compounds exhibited moderated α-amylase inhibition potential. After enzyme inhibition studies compounds were subjected to docking with protein receptors of α-amylase. Docking results were interpreted in terms of binding energies, number of binding interactions, binding amino acids and distance of ligand with the protein during interactions and results of interactions are presented in Fig. 3 and Table 2. Among these ligands 6a and 6d showed the strongest interaction with the binding energy −6.1 and −1.1 kcal mol^−1^ respectively. 6a compound showed dual interaction at position SER-4 and THR-6 while 6d showed one interaction at position ARG-420 with bond distance ranging from 2.8 to 3.0 Å (Table 2). Rest of the compounds also exhibited no interaction. Overall, these compounds represent positive interactions of compounds with α-amylase providing the evidence of their *in vitro* enzyme inhibition potential (Fig. 3). Inhibition of glucosidase and amylase is crucial in the management of type 2 diabetes. Both synthesized and naturally occurring derivatives of heterocyclic molecules have effective biological effects. They have thus far shown strong inhibitory effect against amylase and glucosidase were discovered to be flexible instruments for the creation of novel anti-diabetic drugs.^43^

**Fig. 4:**
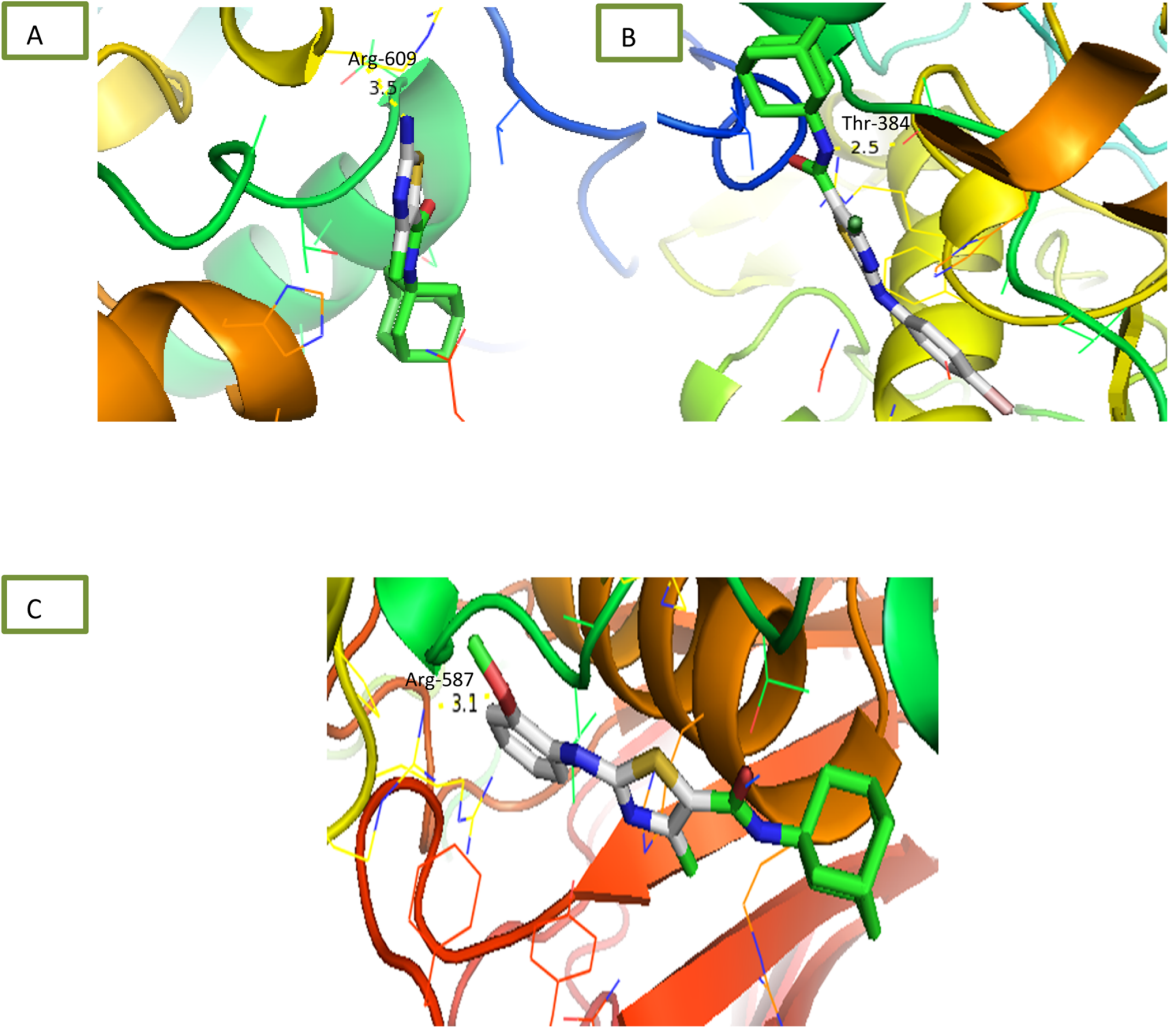
Interaction of α-glucosidase with (A) 6a (B) 6c (C) 6e.

##### 2.2.1.5 Urease inhibition assay

**Fig. 5:**
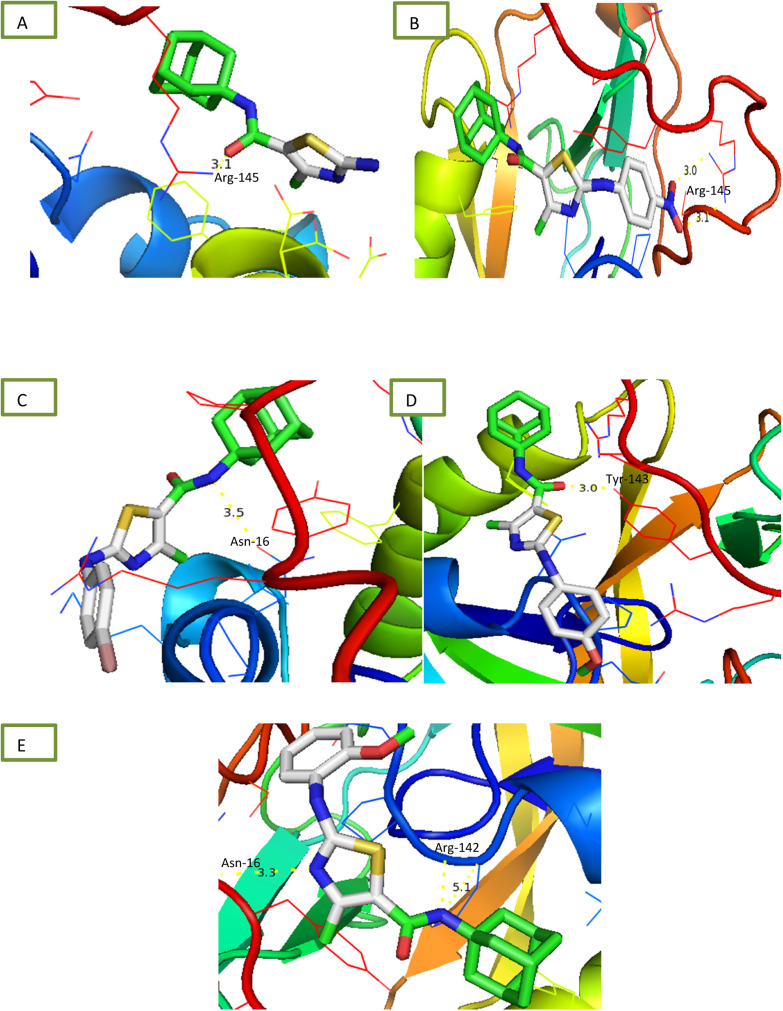
Interaction of urease with (A) 6a (B) 6b (C) 6c (D) 6d (E) 6e.

All compounds were tested for their ability to inhibit urease and results of percentage inhibition are presented in the form of IC_50_ values presented in Table 1. Thiourea used as positive control which revealed prominent enzyme inhibition activity with IC_50_ value 32.76 ± 0.28 μM. The results of IC_50_ represented that the highest activity was shown by the compound 6d with IC_50_ value 32.76 ± 0.28 μM. Compounds, 6g and 6i exhibited low activity with IC_50_ values greater than 100 μM. Other compounds of the series 6a, 6b, 6c, 6e and 6h exhibited moderate activity with IC_50_ value in range of 50 μM to 80 μM. Overall, compounds exhibited good urease activity. After enzyme inhibition studies compounds were subjected to docking with protein receptors of urease. Docking results are presented in Fig. 5 and Table 2 in terms of binding energies, number of binding interactions and distance. All these compounds, 6a, 6b, 6c, 6d and 6e showed the strongest interaction with the binding energy −5.2, −6.1, −5.6, −5.6 and −6.1 kcal mol^−1^ respectively. All mentioned compounds except 6b and 6e showed 1 interaction with amino acids ARG-145, ARG-141, ASN-16 and TYR-143 respectively. The bond distance values range from 3.0 to 5.1 Å (Table 2). Compound, 6b and 6e exhibited double interaction at position ARG-141, ASN-16 and ARG-142 having bond distance between 3.3 to 5.1 Å with bond energy range between −8.9 to −10.1 kcal mol^−1^. Overall, these compounds represent strong positive interactions of compounds with urease providing linkage with *in vitro* activity. Urease is a key target for the treatment of bacterial infections linked to urease that cause kidney stones, UTI, and stomach ulcers. Only a small number of the several compounds that have been identified to be urease inhibitors have made it to clinical trials. New appropriate lead compounds are necessary for the creation of new powerful urease inhibitors.^44^

#### 2.2.2 Biological assay

##### 2.2.2.1 Antibacterial assay

Disc diffusion assay was used to determine the antibacterial potential of newly synthesised compounds as reported earlier.^45^ For experiment, 1% inoculum of *Bacillus subtilis*, *Staphylococcus aureus*, *Escherichia coli* and *Pseudomonas aeruginosa* was transferred to autoclaved nutrient agar media (23 g l^−1^) respectively. Then media were poured in respective plates and allowed to solidify. Samples (100 μM) and Kanamycin drug (10 μM) was loaded on sterile paper disc (5 mm) and these discs were placed on Petriplates at appropriate distance. Plates were incubated for 24 hours at 37 °C and zone of inhibition were measured using Vernier calliper.

#### 2.2.3 DPPH assay

DPPH assay as reported earlier was used to determine the antioxidant potential of newly synthesised compound.^46^ For experiment, 195 DPPH (100 μM) solution was mixed with 5 μl of sample compounds and ascorbic acid (positive control) at concentration of 100, 50 and 25 μM in 96-well plate respectively. Plate was incubated in dark for 30 minutes at 37 °C and then absorbance was measured at 515 nm using Elx800 microtiter plate reader. The experiment was performed in triplicate and percentage scavenging was calculated as following:Percentage scavenging (%) = [1 − absorbance of sample/absorbance of control] × 100

##### 2.2.3.1 α-Glucosidase inhibition assay

Inhibitory α-glucosidase activities were determined in a 96-well microtiter plate based on *p*-nitrophenyl-α-d-glucopyranoside (PNPG) as a substrate.^47^ The α-glucosidase enzyme solution (2 μl), 10 μl PNPG substrate solution (20 mM in phosphate buffer), test sample (5 μl) or acarbose (positive control) at concentration of 100, 50 and 25 μM and buffer (68 μl) were added and mixed in a 96 well microtiter plate and incubated at 37 °C for thirty min. Then after incubation 100 μl of 0.5 mM sodium bicarbonate solution was added to stop the reaction and absorbance was measured at the wavelength of 405 nm with a microtiter plate reader. The experiments were performed in triplicate and percentage inhibition was calculated using following formula and IC_50_ was calculated using GraphPad PrismV8.% inhibition = (absorbance of control − absorbance of sample/absorbance of control) × 100

##### 2.2.3.2 α-Amylase inhibition assay

α-Amylase inhibition assay was performed to calculate the enzyme inhibition potential of newly synthesised compounds.^48^ For experiment, 40 μl starch (0.5 mg ml^−1^), 10 μl enzyme (0.1 U), 40 μl phosphate buffer (pH 6.8) and 10 μl sample compounds and acarbose (positive control) at concentration of 100, 50 and 25 μM in 96-well plate respectively. In blank 40 μl starch and 50 μl phosphate buffer was added. Plate was incubated for 30 minutes at 50 °C followed by addition of 20 μl HCL (1 M) as stopping reagent and 100 μl of iodine reagent (5 mM KI + 5 mM I_2_). Then absorbance was measured at 540 nm using Elx800 microtiter plate reader. The experiment was performed in triplicate and percentage inhibition was calculated using following formula and IC_50_ was calculated using GraphPad PrismV8.% inhibition = 100 − ((absorbance of blank − absorbance of sample/absorbance of blank) × 100)

##### 2.2.3.3 Urease inhibition activity

The antiurease activity of the compounds was measured by determining the amount of free ammonia produced as described earlier.^49^ The experiment was performed by mixing 10 μl enzyme (0.1 U per reaction), 30 μl of each concentration (100, 50 and 25 μM) of compound and 50 μl buffer of pH 8.2 consisting of 100 mM urea, 0.01 M LiCl_2_, 1 mM EDTA and 0.01 M K_2_HPO_4_. Reaction mixtures were incubated at 37 °C for 15 min in 96-well plate. Then 50 μl of phenol reagent (0.005% sodium nitroprusside and 1% phenol) and 50 μl of alkali reagent (0.1% NaOCl and 0.5% NaOH and) were added to each well and plates incubated at 37 °C for 50 min. Assay was performed in triplicate and absorbance was recoded at 625 nm using microplate reader. The antiurease activity of the compounds were calculated in percentage using following formula and IC_50_ was calculated using GraphPad PrismV8.% inhibition = (absorbance of control − absorbance of sample/absorbance of control) × 100

### 2.3 Docking studies

Comparative docking analysis of minimized protein structures were performed with α-amylase, urease and α-glucosidase. Each ligand–receptor complex was subjected to careful analysis for ideal docked poses on the basis of least binding energy scores and maximum number of cluster conformations. Binding energy values of stable docked conformations are shown below. Positioning of ligands onto the surface of α-amylase, urease and α-glucosidase were keenly monitored to explore the binding pocket dynamics and residual contributions of each protein in association with docked ligand. The detailed residual contributions of individual complexes are shown below. Generally, structural insights of inhibitor binding to α-amylase, urease and α-glucosidase revealed predominant contributions of hydrophobic residues lying in the periphery of active sites.

The ligand was developed by using ChemDraw from which it was converted to SDF file. The receptors alpha amylase, alpha glucosidase and urease were downloaded from RCSB site as PDB files having codes 2QMK, 5NN3 and 4AC7 respectively. These receptors and ligand files were prepared by software BIOVIA Discovery Studio Client 2020 and MGL Tools 1.5.6 and saved as PDBQT file. The docking was performed *via* AutoDock Vina. The results were analysed by PyMOL where binding residues and bond lengths were noted.^50^

#### 2.3.1 Docking results

The results are shown in table below. 6a showed the lowest energy −6.71 kcal mol^−1^ for alpha amylase. 6a showed lowest energy −3.6 kcal mol^−1^ for alpha-glucosidase. 6b and 6e showed the lowest −6.1 kcal mol^−1^ for urease.

## 3. Structure activity relationship

**Fig. 6:**
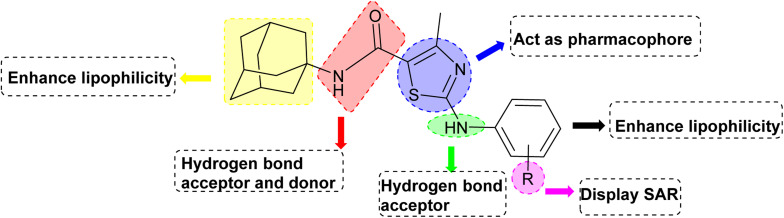
Active sites in synthesized compounds for potent biological activity.

Structure Activity Relationships (SAR) are relations between the molecular structure and biological or physicochemical activity of chemicals. The SAR allows to design and modify the structure to obtain effective drugs. A series of adamantyl hybrid substituted phenyl thiazoles was designed and synthesized as potent inhibitors of urease, amylase and glucosidase. The pharmacophore of these hybrids comprised of various active sites (Fig. 6).

**Fig. 7:**
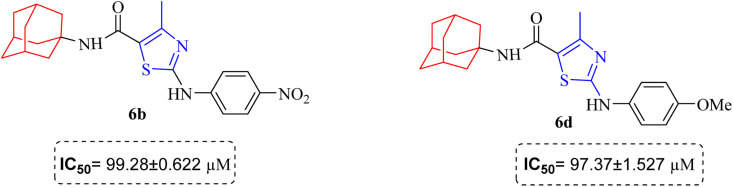
Compounds 6b and 6d along with IC_50_ values indicating *para* substituted compounds are more potent.

The results inhibitory effects for α-amylase of compounds revealed that the compound 6d was found to most potent in the series and exhibited the lowest IC_50_ values amongst the other compounds of series hence more active. In compound 6d, the methoxy group at *para*-position could facilitate to occupy the whole pocket and lead to stronger hydrophobic interactions. The second most active compound for α-amylase was 6b because of presence of (–NO_2_) group at *para* position (Fig. 7). The results showed that the *para* position showed the most effective results both with withdrawing and donating groups.

**Fig. 8:**
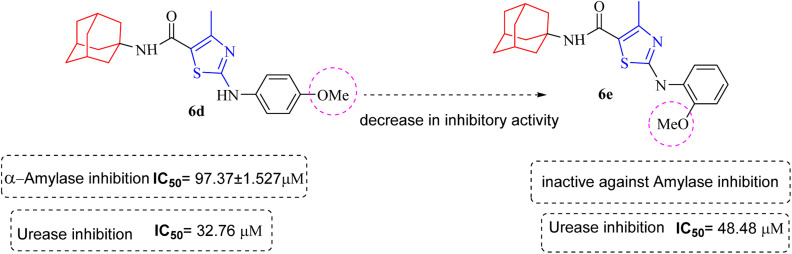
Structural variation from 6d to 6e showing decrease in amylase and urease inhibitory action.

Similarly compound 6d was also found to be most active for inhibitory affects for urease due to the presence of methoxy group at *para* position (Fig. 8). The results indicated that electron donating groups at *para* position were most active than other substituents at phenyl ring. However, potency is markedly reduced when electron donating groups were replaced with the withdrawing groups at the same position. For both urease and amylase activity 6d was the most potent and inhibitory affect decreased when the position of methoxy group was changed from *para* to *ortho* due to intramolecular hydrogen bonding with NH.

**Fig. 9:**
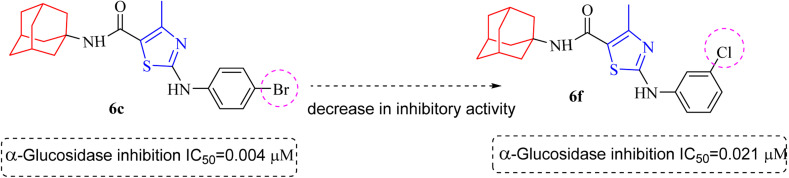
Structural variation from 6c to 6f indicating decrease in α-glucosidase inhibition.

Compound 6c was found to be most potent for inhibitory effects of α-glucosidase. The bromo group at *para* position resulted in a slight enhancement of activity in comparison to 6f with chloro group at *meta* position indicating that the halogen bromine at *para* position is more favorable for interaction than chlorine (Fig. 9).

The compound 6h bears nimesulide group which creates crowding/steric hindrance and was found to be only moderately active for urease and showed no activity for others.

## 4. Conclusions

In this research, we synthesized a small library of new amantadine clubbed *N*-aryl amino thiazole derivatives (6a–j) with structural variation at the nitrogen atom of the amino thiazole moiety. We then investigated their effects against urease, α-glucosidase, and α-amylase enzymes by determining their levels of IC_50_ values. Our results showed that all the compounds inhibited all three metabolic enzymes, with compound (6a) being particularly effective against urease and both digestive enzymes. Compounds (6a–e) were found to be effective against the urease enzyme. The different moieties of the new derivatives were found to be essential for inhibiting specific enzyme receptors. These substances were found to have a significantly stronger inhibitory effect than standard drugs. Molecular docking studies also showed that the selected compounds interact significantly within the active pocket of the urease enzyme, as well as α-amylase and α-glucosidase, and demonstrate convincing inhibition capacity for these enzymes. Our findings suggest that these synthesized substances may be potential candidates for the design of new drugs in the development and synthesis of inhibitors for metabolic enzymes used in the therapy of various diseases such as epilepsy, obesity, glaucoma, cancer, and type 2 diabetes in the future.

## 5. Experimental

### 5.1 Reagents and equipment

Analytical grade reagents and chemicals were employed throughout the experimental procedures. Solvent purification and drying were carried out using standard protocols. The *R*_f_ values were determined using pre-coated silica gel plates Kiesel 60 F_254_ by observing under the UV. Melting points were determined by using Stuart SMP_3_ digital melting point apparatus *via* open-end capillary. ^1^H and ^13^C NMR spectra were recorded on Bruker 300/600 MHz and 75/150 MHz spectrophotometer, respectively, using DMSO as a solvent. Functional group detection through IR spectra was done on a PerkinElmer spectrometer using KBr pellets.

### 5.2 General procedure for the synthesis of amantadine-based *N*-aryl amino thiazoles

*N*-Adamantyl-3-oxobutanamide (3) was formed by refluxing amantadine (0.5 g, 1 mmol) (1) and methyl acetoacetate (2) in a round bottom flask under solvent-free conditions. The reaction mixture was refluxed for 3 hours. The reaction was monitored by TLC. The product was extracted from the reaction mixture by solvent extraction with ethyl acetate. The ethyl acetate layer was collected, and the solvent was evaporated under reduced pressure to get the required product (3).

The mixture was further dissolved in ethanol (10 ml) followed by dropwise addition of bromine (1.2 mmol) diluted with acetic acid, under controlled conditions at 0 °C, and stirred for 20 min resulting in the appearance of white precipitates. The white solid was then filtered off and washed with water and recrystallized in ethanol to afford *N*-adamantyl-2-bromo-3-oxobutanamide (4). white solid; yield: 81%; melting point: 150–153 °C; *R*_f_: 0.5 (*n*-hexane : ethyl acetate: 1 : 9) ^1^H-NMR (300 MHz, DMSO-*d*_6_) *δ* (ppm): 8.15 (s, 1H, N–H, amide), 2.96 (s, 3H, CH_3_), 5.17 (s, 1H, CH), 1.63–2.02 (m, 15H, adamantyl), ^13^C-NMR (75 MHz, DMSO-*d*_6_) *δ* (ppm): 197.0, 163.9, 55.3, 52.0, 36.2, 29.1, 26.8; GC-MS calcd for C_14_H_20_BrNO_2_ [M + H]^+^ 314.07, found 314.07; elemental analysis: C, 53.51; H, 6.42; Br, 25.43; N, 4.46; O, 10.18.

The cyclization was carried out by the reaction of prepared precursor *N*-(adamantyl)-2-bromo-3-oxobutanamide (1 mmol) (4) with synthesized hydrolyzed phenyl thioureas (1 mmol) (5a–j). Ethanol (5 ml) was used as a solvent, taken in round bottom flask along with reactants and refluxed for 2 hours. Precipitates formed upon aqueous workup were filtered and collected. The desired products were purified *via* recrystallization in ethanol to yield amantadine-based *N*-aryl amino thiazoles (6a–j).

### 5.3 Experimental data

#### 5.3.1 *N*-(Adamantan-1-yl)-2-amino-4-methylthiazole-5-carboxamide (6a)


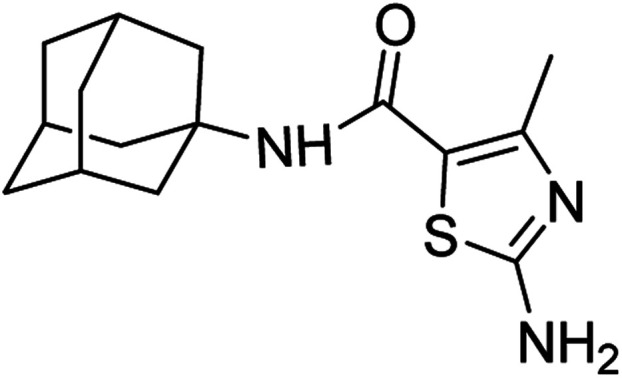
White solid; yield: 79%; melting point: 256–257 °C; *R*_f_: 0.4 (*n*-hexane : ethyl acetate: 2 : 3); ^1^H-NMR (400 MHz, DMSO-*d*_6_) *δ* (ppm): 7.22 (s, 2H, NH_2_), 6.63 (s, 1H, NH), 2.27 (s, 3H, CH_3_), 2.05–1.97 (m, 9H), 1.63 (t, *J* = 2.9 Hz, 6H). ^13^C-NMR (101 MHz, DMSO-*d*_6_) *δ* (ppm): 167.25 (C=O, amide), 161.46, 151.01 (C=N: thiazole), 114.70, (adamantyl: 51.47, 41.03, 36.04, 28.84), 17.07 (CH_3_). GC-MS calcd for C_15_H_21_N_3_OS [M + H]^+^ 291.14, found 291.0; elemental analysis: C, 61.82; H, 7.26; N, 14.42; O, 5.49; S, 11.00.

#### 5.3.2 *N*-(Adamantan-1-yl)-4-methyl-2-((4-nitrophenyl)amino)thiazole-5-carboxamide (6b)


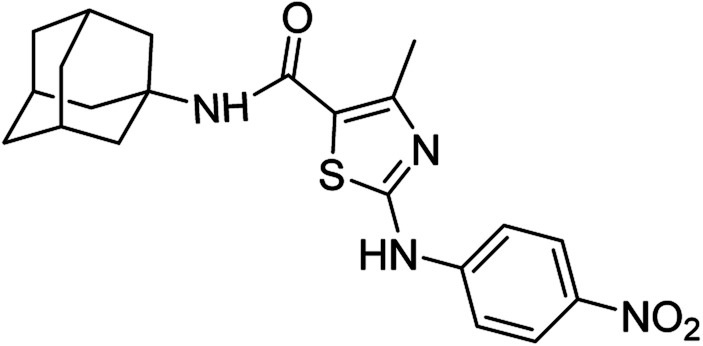
Green solid; yield: 75%; melting point: 300–303 °C; *R*_f_: 0.2 (*n*-hexane : ethyl acetate: 2 : 3); ^1^H-NMR (300 MHz, DMSO-*d*_6_) *δ* (ppm): 11.13 (s, 1H, N–H), 7.25 (s, 1H, N–H, amide), 2.45 (s, 3H, CH_3_), 1.63–2.02 (m, 15H, adamantyl), 7.81–7.84 (d, 2H, aromatic), 8.21–8.46 (d, 2H, *J* = 8.7 Hz aromatic) ^13^C-NMR (75 MHz, DMSO-*d*_6_) *δ* (ppm): 161.1 (C=O, amide), 161.0, 150.5 (C=N: thiazole), 146.9, 140.9, 125.9, 119.4, 116.9, (adamantyl: 52.3, 41.3, 36.4, 29.3), 17.7 (CH_3_). Elemental analysis: C, 61.15; H, 5.86; N, 13.58; O, 11.64; S, 7.77.

#### 5.3.3 *N*-(Adamantan-1-yl)-4-methyl-2-((4-bromophenyl)amino)thiazole-5-carboxamide (6c)


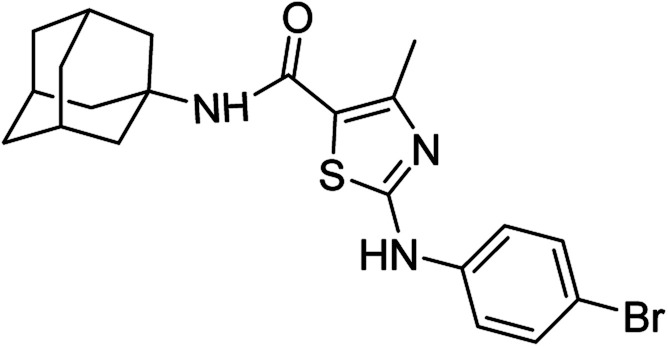
White solid; yield: 74%; melting point: 277–280 °C; *R*_f_: 0.6 (*n*-hexane : ethyl acetate: 2 : 3); ^1^H-NMR (300 MHz, DMSO-*d*_6_) *δ* (ppm): 10.48 (s, 1H, N–H), 7.06 (s, 1H, N–H, amide), 2.50 (s, 3H, CH_3_), 1.63–2.02 (m, 15H, adamantyl), 7.46–7.49 (d, 2H, aromatic), 7.56–7.59 (d, 2H, aromatic) ^13^C-NMR (75 MHz, DMSO-*d*_6_) *δ* (ppm): 162.0 (C=O, amide), 161.4, 150.7 (C=N: thiazole), 140.4, 132.1, 119.5, 113.3, (adamantyl: 52.3, 41.3, 36.4, 29.3), 17.7 (CH_3_). Elemental analysis: C, 56.50; H, 5.42; Br, 17.90; N, 9.41; O, 3.58; S, 7.18.

#### 5.3.4 *N*-(Adamantan-1-yl)-4-methyl-2-((4-methoxyphenyl)amino)thiazole-5-carboxamide (6d)


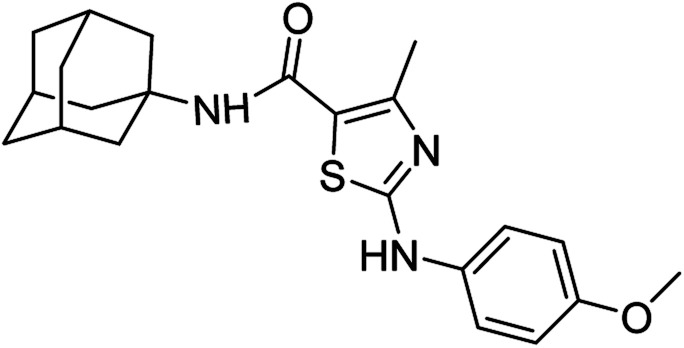
White solid; yield: 79%; melting point: 226–229 °C; *R*_f_: 0.5 (*n*-hexane : ethyl acetate: 2 : 3); ^1^H-NMR (300 MHz, DMSO-*d*_6_) *δ* (ppm): 10.11 (s, 1H, N–H), 6.99 (s, 1H, N–H, amide), 2.50 (s, 3H, CH_3_), 3.72 (s, 3H, OCH_3_), 1.63–2.02 (m, 15H, adamantyl), 6.89–6.92 (d, 2H, aromatic), 7.44–7.47 (d, 2H, aromatic) ^13^C-NMR (75 MHz, DMSO-*d*_6_) *δ* (ppm): 163.5 (C=O, amide), 161.7, 155.1 (C=N: thiazole), 151.2, 134.4, 120.0, 115.8, 114.7, 55.6 (CH_3_), (adamantyl: 52.3, 41.3, 36.4, 29.3), 17.7 (CH_3_). LC-MS calcd for C_22_H_27_N_3_O_2_S [M + H]^+^ 397.18, found 397.0; elemental analysis: C, 66.47; H, 6.85; N, 10.57; O, 8.05; S, 8.06.

#### 5.3.5 *N*-(Adamantan-1-yl)-4-methyl-2-((2-methoxyphenyl)amino)thiazole-5-carboxamide (6e)


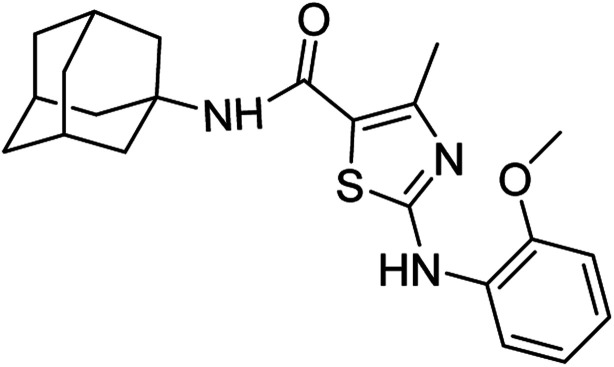
White solid; yield: 77%; melting point: 210–212 °C; *R*_f_: 0.6 (*n*-hexane : ethyl acetate: 2 : 3); ^1^H-NMR (300 MHz, DMSO-*d*_6_) *δ* (ppm): 9.60 (s, 1H, N–H), 6.96 (s, 1H, N–H, amide), 2.49 (s, 3H, CH_3_), 1.63–2.02 (m, 15H, adamantyl), 6.90–8.21 (m, 4H, aromatic) ^13^C-NMR (75 MHz, DMSO-*d*_6_) *δ* (ppm): 163.5 (C=O, amide), 161.7, 150.4 (C=N: thiazole), 149.2, 130.0, 123.4, 120.9, 119.9, 117.2, 111.5, 56.1 (CH_3_), (adamantyl: 52.3, 41.3, 36.4, 29.3), 17.7 (CH_3_). Elemental analysis: C, 66.47; H, 6.85; N, 10.57; O, 8.05; S, 8.06.

#### 5.3.6 *N*-(Adamantan-1-yl)-4-methyl-2-((3-chlorophenyl)amino)thiazole-5-carboxamide (6f)


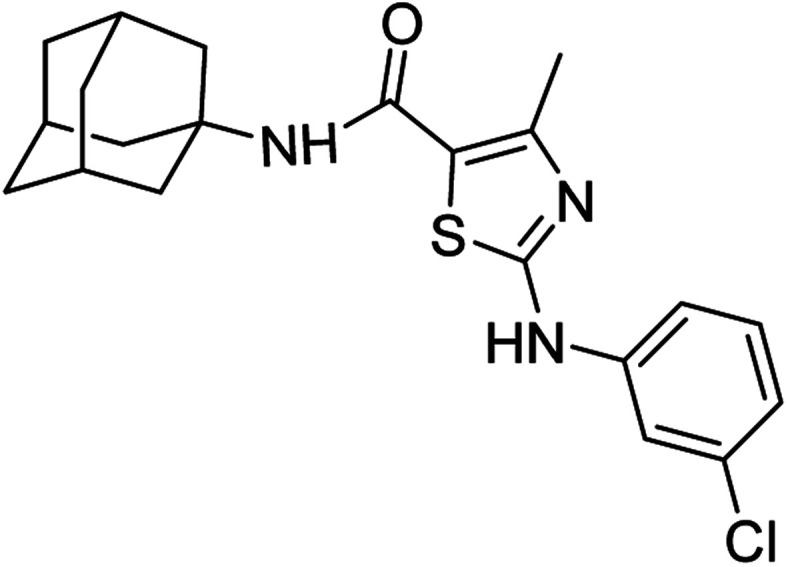
White solid; yield: 79%; melting point: 253–254 °C; *R*_f_: 0.5 (*n*-hexane : ethyl acetate: 2 : 3); ^1^H-NMR (300 MHz, DMSO-*d*_6_) *δ* (ppm): 10.54 (s, 1H, N–H), 6.99 (s, 1H, N–H, amide), 2.50 (s, 3H, CH_3_), 1.63–2.02 (m, 15H, adamantyl), 7.00–7.84 (m, 4H, aromatic) ^13^C-NMR (75 MHz, DMSO-*d*_6_) *δ* (ppm): 161.9 (C=O, amide), 161.4, 150.6 (C=N: thiazole), 142.4, 133.8, 131.0, 121.6, 117.7, 116.9, 116.0, (adamantyl: 52.3, 41.3, 36.4, 29.3), 17.7 (CH_3_). LC-MS calcd for C_21_H_24_ClN_3_OS [M + H]^+^ 401.3, found 400.0. Elemental analysis: C, 62.75; H, 6.02; Cl, 8.82; N, 10.45; O, 3.98; S, 7.98.

#### 5.3.7 *N*-(Adamantan-1-yl)-4-methyl-2-((2-chlorophenyl)amino)thiazole-5-carboxamide (6g)


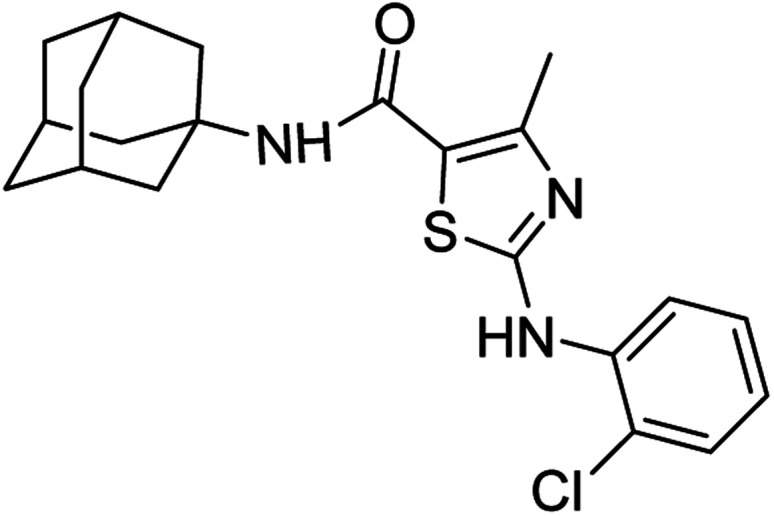
White solid; yield: 80%; melting point: 202–203 °C; *R*_f_: 0.6 (*n*-hexane : ethyl acetate: 2 : 3); ^1^H-NMR (300 MHz, DMSO-*d*_6_) *δ* (ppm): 9.77 (s, 1H, N–H), 6.98 (s, 1H, N–H, amide), 2.36 (s, 3H, CH_3_), 1.63–2.02 (m, 15H, adamantyl), 7.08–8.08 (m, 4H, aromatic) ^13^C-NMR (75 MHz, DMSO-*d*_6_) *δ* (ppm): 163.5 (C=O, amide), 161.5, 150.3 (C=N: thiazole), 137.9, 130.0, 128.3, 125.1, 124.7, 123.3, (adamantyl: 52.3, 41.3, 36.4, 29.3), 17.5 (CH_3_). LC-MS calcd for C_21_H_24_ClN_3_OS [M + H]^+^ 401.3, found 400.2. Elemental analysis: C, 62.75; H, 6.02; Cl, 8.82; N, 10.45; O, 3.98; S, 7.98.

#### 5.3.8 *N*-Adamantyl-4-methyl-2-((4-(methylsulfonamido)-3-phenoxyphenyl)amino) thiazole-5-carboxamide (6h)


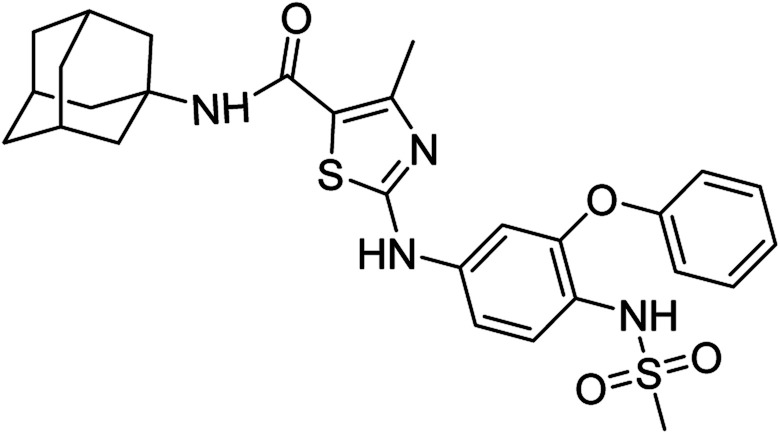
White solid; yield: 75%; melting point: 156–160 °C; *R*_f_: 0.2 (*n*-hexane : ethyl acetate: 2 : 3); ^1^H-NMR (300 MHz, DMSO-*d*_6_) *δ* (ppm): 10.37 (s, 1H, N–H), 9.18 (s, 1H, N–H, sulfonamide), 7.00 (s, 1H, N–H, amide), 2.95 (s, 3H, CH_3_, sulfonamide), 2.26 (s, 3H, CH_3_), 1.63–2.20 (m, 15H, adamantyl), 7.11–7.48 (m, 8H, aromatic) ^13^C-NMR (75 MHz, DMSO-*d*_6_) *δ* (ppm): 161.9 (C=O, amide), 161.4, 156.01 (C=N: thiazole), 152.6, 150.5,140.3, 130.5, 129.3, 124.6, 120.9, 120.3, 117.3, 112.1, 106.8, (adamantyl: 52.2, 41.3, 36.4, 29.3), 17.5 (CH_3_). Elemental analysis: C, 60.85; H, 5.84; N, 10.14; O, 11.58; S, 11.60.

#### 5.3.9 *N*-Adamantyl-2-((3-fluorophenyl)amino)-4-methylthiazole-5-carboxamide (6i)


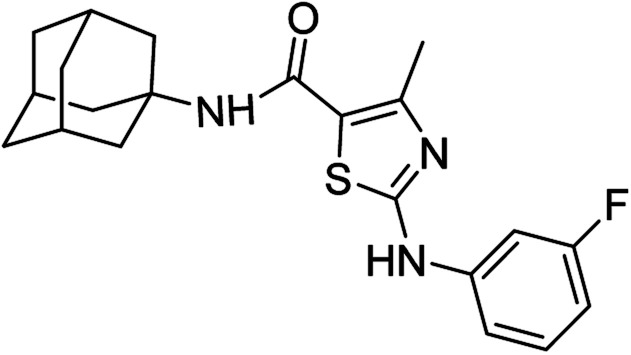
White solid; yield: 78%; melting point: 220–225 °C; *R*_f_: 0.4 (*n*-hexane : ethyl acetate: 2 : 3); ^1^H-NMR (300 MHz, DMSO-*d*_6_) *δ* (ppm): 10.37 (s, 1H, N–H), 9.18 (s, 1H, N–H, sulfonamide), 7.00 (s, 1H, N–H, amide), 2.50 (s, 3H, CH_3_, sulfonamide), 1.63–2.20 (m, 15H, adamantyl), 7.36–7.87 (m, 4H, aromatic) ^13^C-NMR (75 MHz, DMSO-*d*_6_) *δ* (ppm): 161.9 (C=O, amide), 161.4, 156.01 (C=N: thiazole), 152.6, 150.5,140.3, 130.5, 129.3, 124.6, 120.9, 120.3, 117.3, 112.1, 106.8, (adamantyl: 52.2, 41.3, 36.4, 29.3), 17.5 (CH_3_). Elemental analysis: C, 65.43; H, 6.28; F, 4.93; N, 10.90; O, 4.15; S, 8.32.

#### 5.3.10 *N*-Adamantanyl-2-(((3s,5s,7s)-adamantan-1-yl)amino)-4-methylthiazole-5-carboxamide (6j)


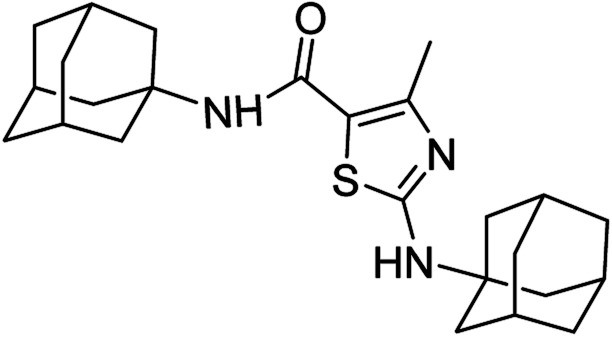
White solid; yield: 75%; melting point: 250–253 °C; *R*_f_: 0.3 (*n*-hexane : ethyl acetate: 2 : 3); ^1^H-NMR (300 MHz, DMSO-*d*_6_) *δ* (ppm): 7.50 (s, 1H, N–H, amide), 6.80 ppm (s, 1H, N–H), 2.50 (s, 3H, CH_3_, sulfonamide), 1.63–2.28 (m, 30H, adamantyl), ^13^C-NMR (75 MHz, DMSO-*d*_6_) *δ* (ppm): 165.09 (C=O, amide), 162.08, 151.07 (C=N: thiazole), 114.5, (adamantyl: 53.21, 51.97, 41.51, 41.3, 36.49, 36.35, 29.3), 17.86 (CH_3_). Elemental analysis: C, 70.55; H, 8.29; N, 9.87; O, 3.76; S, 7.53.

## Conflicts of interest

There are no conflicts to declare.

## Supplementary Material

RA-013-D3RA05330J-s001
