# Supplementary material for: Synthesis of amantadine clubbed N-aryl amino thiazoles as potent urease, α-amylase & α-glucosidase inhibitors, kinetic and molecular docking studies
Source: RSC Adv. 2023 Aug 22;13(36):24988–5001. doi: 10.1039/d3ra05330j (PMC10442672; doi:10.1039/d3ra05330j)
Supplement: RA-013-D3RA05330J-s001 [file RA-013-D3RA05330J-s001.pdf]

Electronic Supplementary Material (ESI) for RSC Advances.
This journal is © The Royal Society of Chemistry 2023

Synthesis of Amantadine clubbed *N*-Aryl amino thiazoles as potent Urease, α-Amylase & α-Glucosidase inhibitors and their kinetic and molecular docking studies.

Synthesis of Amantadine clubbed *N*-Aryl amino thiazoles as potent Urease, α-Amylase & α-Glucosidase inhibitors, kinetic and molecular docking studies.

Fatima Tuz Zahra[a], Aamer Saeed*[a], Atteeque Ahmed[a], Hammad Ismail[b], Muhammad Umar Ijaz[c], Fernando Albericio,*[d,e]

[a] *Department of Chemistry Quaid-i-Azam University-45320, Islamabad, Pakistan*

[b]*Department of Biochemistry and Biotechnology, University of Gujrat, Gujrat, 50700, Pakistan*

[c]*Department of Zoology, Wildlife and Fisheries, University of Agriculture, Faisalabad 38040, Pakistan*

[d] *Peptides Science Laboratory, School of Chemistry and Physics, University of KwaZulu-Natal, Westville, Durban 4000, South Africa.*

[e]*CIBER-BBN, Networking Centre on Bioengineering, Biomaterials and Nanomedicine, and Department of Organic Chemistry, University of Barcelona, 08028 Barcelona, Spain.*

Correspondence
Prof. Dr. Aamer Saeed
Email: asaeed@qau.edu.pk ; ORCID: http://orcid.org/0000-0002-7112-9296
Contact number: +92-51-9064-2128, Fax: +92-51-9064-2241

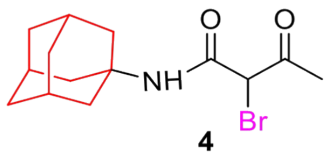

4

Chemical Formula: $C_{14}H_{20}BrNO_2$
Exact Mass: 313.07
Molecular Weight: 314.22
m/z: 313.07 (100.0%), 315.07 (97.3%), 314.07 (15.1%), 316.07 (14.7%), 315.07 (1.1%), 317.07 (1.0%)
Elemental Analysis: C, 53.51; H, 6.42; Br, 25.43; N, 4.46; O, 10.18

Fragmentation pattern:

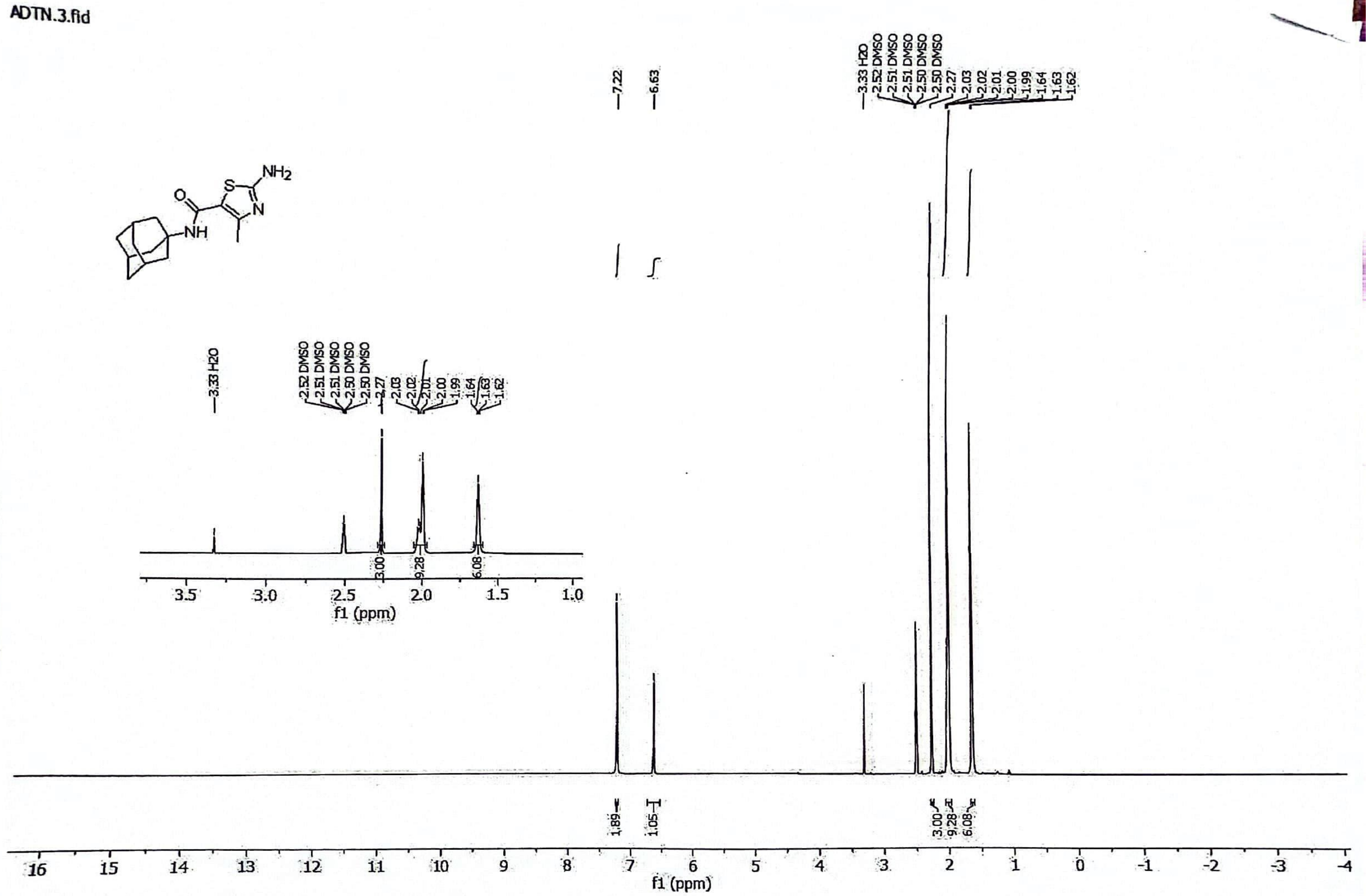
ADTN.3.fid
NH2
S
N
O
NH
7.22
6.63
3.33 H2O
2.52 DMSO
2.51 DMSO
2.51 DMSO
2.50 DMSO
2.50 DMSO
2.27
2.03
2.02
2.01
2.00
1.99
1.64
1.63
1.62
3.00
9.28
6.08
3.5
3.0
2.5
2.0
1.5
1.0
f1 (ppm)
1.89
1.05
16
15
14
13
12
11
10
9
8
7
6
5
4
3
2
1
0
-1
-2
-3
-4
f1 (ppm)

ADTN.4.fid

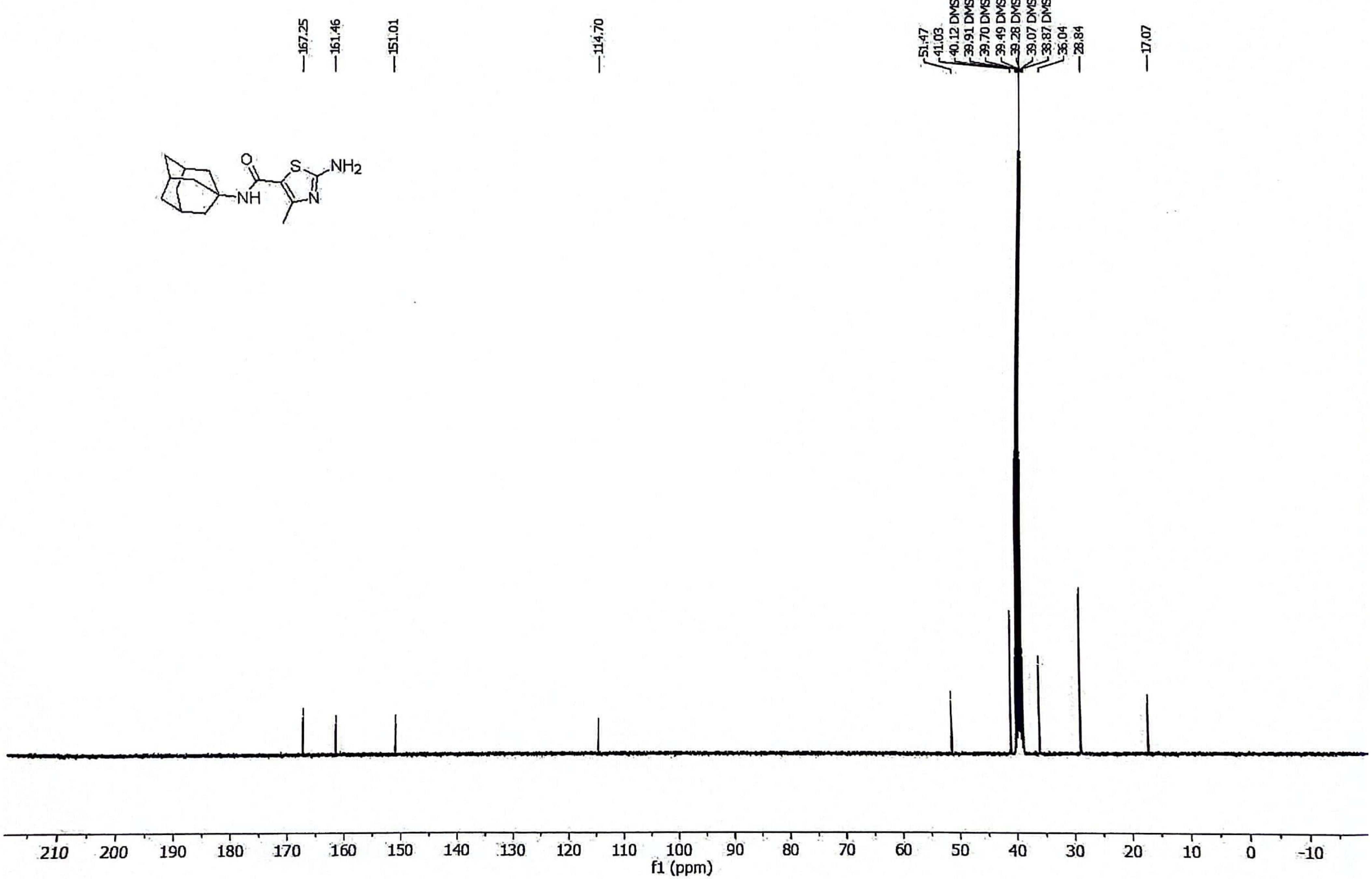
167.25
161.46
151.01
114.70
51.47
41.03
40.12 DMSO
39.91 DMSO
39.70 DMSO
39.49 DMSO
39.28 DMSO
39.07 DMSO
38.87 DMSO
36.04
28.84
17.07
O
S
NH2
NH
N
210 200 190 180 170 160 150 140 130 120 110 100 90 80 70 60 50 40 30 20 10 0 -10
f1 (ppm)

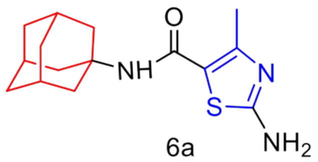

6a

Chemical Formula: $C_{15}H_{21}N_3OS$

Exact Mass: 291.14

Molecular Weight: 291.41

m/z: **291.14** (100.0%), 292.14 (16.2%), 293.14 (4.5%), 293.15 (1.2%), 292.14 (1.1%)

Elemental Analysis: C, 61.82; H, 7.26; N, 14.42; O, 5.49; S, 11.00

Fragmentation pattern:

DR.AAMER SAEED/FATIMA/TH-11_13CNMR_DMSO

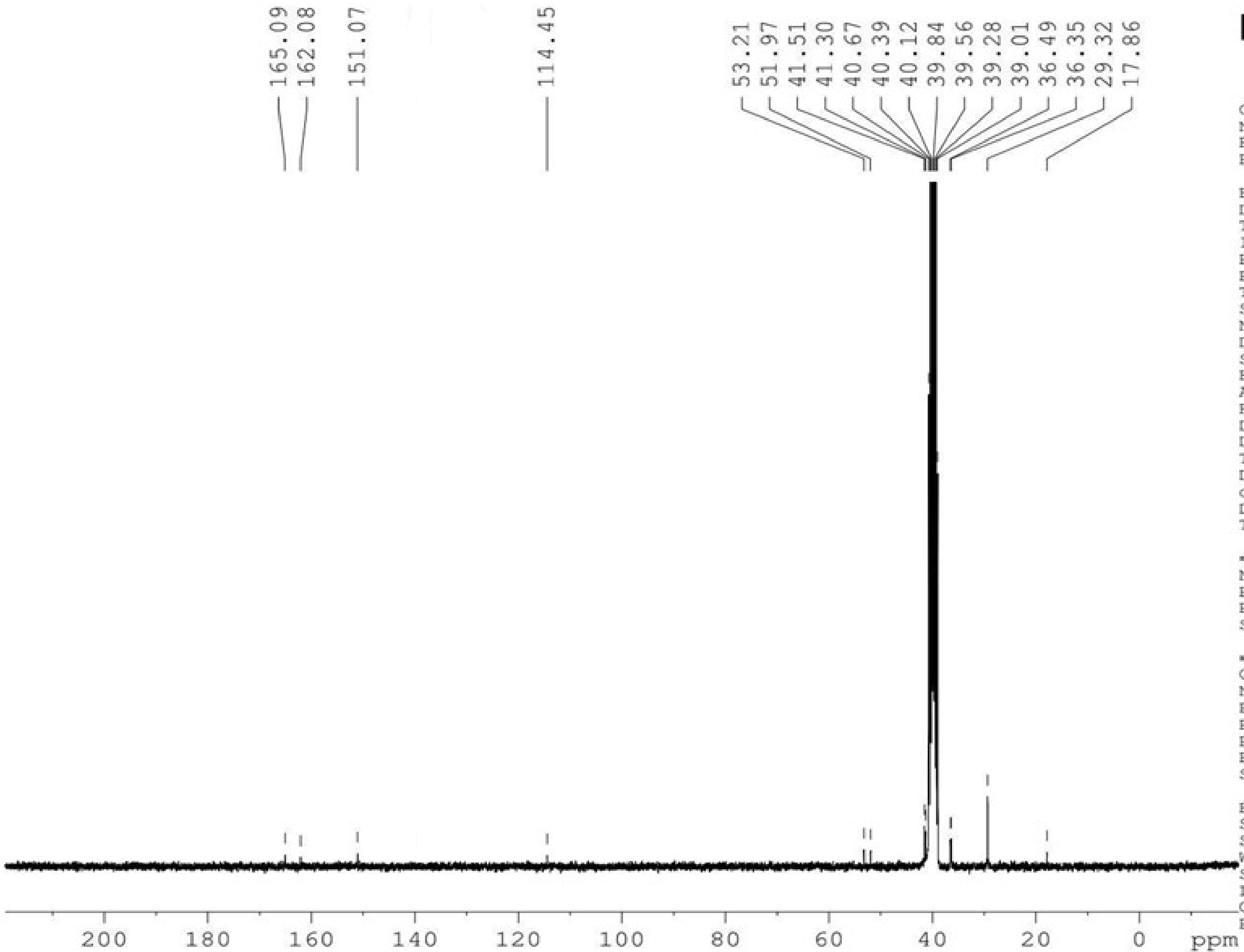

BRUKER

Current Data Parameters
NAME TH-11_13CNMR_DMSO
EXPNO 1
PROCNO 1

F2 - Acquisition Parameters
Date_ 20230619
Time 9.30
INSTRUM spect
PROBHD 5 mm BBO BB-1H
PULPROG zgpg30
TD 35968
SOLVENT DMSO
NS 18432
DS 0
SWH 17985.611 Hz
FIDRES 0.500045 Hz
AQ 0.9999604 sec
RG 32768
DW 27.800 usec
DE 6.00 usec
TE 298.2 K
D1 2.00000000 sec
d11 0.03000000 sec
DELTA 1.89999998 sec
TD0 1

======== CHANNEL f1 ========
NUC1 13C
P1 6.00 usec
PL1 -5.00 dB
SFO1 75.4752953 MHz

======== CHANNEL f2 ========
CPDPRG2 waltz16
NUC2 1H
PCPD2 80.00 usec
PL2 2.00 dB
PL12 20.98 dB
PL13 20.00 dB
SFO2 300.1312005 MHz

F2 - Processing parameters
SI 32768
SF 75.4677490 MHz
WDW EM
SSB 0
LB 1.00 Hz
GB 0
PC 1.40
